# Weighted single step GWAS reveals genomic regions associated with economic traits in Murrah buffaloes

**DOI:** 10.1080/10495398.2024.2319622

**Published:** 2024-03-04

**Authors:** Linda George, Rani Alex, Gopal Gowane, Vikas Vohra, Pooja Joshi, Ravi Kumar, Archana Verma

**Affiliations:** National Dairy Research Institute, Karnal, India

**Keywords:** WssGWAS, ssGBLUP, Murrah buffalo, ddRAD data

## Abstract

The objective of the present study was to identify genomic regions influencing economic traits in Murrah buffaloes using weighted single step Genome Wide Association Analysis (WssGWAS). Data on 2000 animals, out of which 120 were genotyped using a double digest Restriction site Associated DNA (ddRAD) sequencing approach. The phenotypic data were collected from NDRI, India, on growth traits, viz., body weight at 6M (month), 12M, 18M and 24M, production traits like 305D (day) milk yield, lactation length (LL) and dry period (DP) and reproduction traits like age at first calving (AFC), calving interval (CI) and first service period (FSP). The biallelic genotypic data consisted of 49353 markers post-quality check. The heritability estimates were moderate to high, low to moderate, low for growth, production, reproduction traits, respectively. Important genomic regions explaining more than 0.5% of the total additive genetic variance explained by 30 adjacent SNPs were selected for further analysis of candidate genes. In this study, 105 genomic regions were associated with growth, 35 genomic regions with production and 42 window regions with reproduction traits. Different candidate genes were identified in these genomic regions, of which important are OSBPL8, NAP1L1 for growth, CNTNAP2 for production and ILDR2, TADA1 and POGK for reproduction traits.

## Introduction

The identification of significant SNPs and their genes affecting economically important traits is a vital step to identify major genes and understand the genetic architecture and physiology behind the expression of the phenotypes. Genome wide availability of SNPs from Double digest restriction-site associated sequencing (ddRAD) permits greater tractability and strength in region recovery and a substantial decrease in cost of sequencing. It provides a reduced representation of the sequenced genome to produce a large set of SNP markers, which can be precisely used for genetic diversity, association, population study of large number of samples with less cost.^1^ Genome wide association studies (GWAS) of quantitative or complex traits is a powerful tool to identify the candidate genes for each traits.^2^ In classical GWAS, it consider each SNP marker as a covariate in the model.^3^ The single step GWAS (ssGWAS) consider all available SNPs of genotyped animals jointly with phenotype of all genotyped and non-genotyped animals and use the genetic relationship found in the pedigree for animals that have not been genotyped.^4^ The single-step procedure combines genomic relationships from genotyped individuals with pedigree relationships with non-genotyped individuals, developing a realized relationship matrix (H),^5^ and this integration should allow information on unselected animals to be included, with all relationships tracing back to a conceptual unselected base population. In multistep procedure, computing genomic prediction is complicated, due to creating pseudo-data from phenotypic data of relatives for genotyped animals and suboptimal for GWAS.^6^ Genotyping of animals is still a costly affair for developing and underdeveloped parts of the world. Single-step procedure utilizes minimum genomic data along with available deep pedigree information for genomic prediction.

Economic efficiency of any production system depends on the early stage of development of animal, which are measured from birth to older age. Growth traits are included in selection criteria, as they have moderate to high heritability and moderate genetic correlation between them, which are required for better response to selection.^7^^,^^8^ The lifetime productivity of cattle initiates from the onset of puberty, AFC, duration of service period and inter-calving interval.^9^ For reproduction traits, the response to selection is likely to be slow as a consequence of low heritability and late recording of traits. Irrespective of system of production, herd reproductive performance is a foremost key of profitability. Buffaloes have well defined calving patterns based on season. In these limitations, identification of superior genetic variants associated with reproduction traits will aid in the better performance of buffaloes.

There is shortage of reports in the literature regarding investigations on the genomic estimates of genetic parameters and also identification of marker effects for further association with economic traits of dairy buffaloes. This has occurred mainly due to less number of genotyped animals, non-availability of phenotypic records and higher cost of genotyping of animals. The aim of the current study is to use the single-step procedure to estimate genetic parameters for the economic traits of dairy buffaloes and also to identify the genomic regions influencing growth, reproduction and production traits in Murrah buffaloes using weighted single-step genome wide association studies (WssGWAS).

## Materials and methods

### Genotypic data and quality control

**Figure 1.**
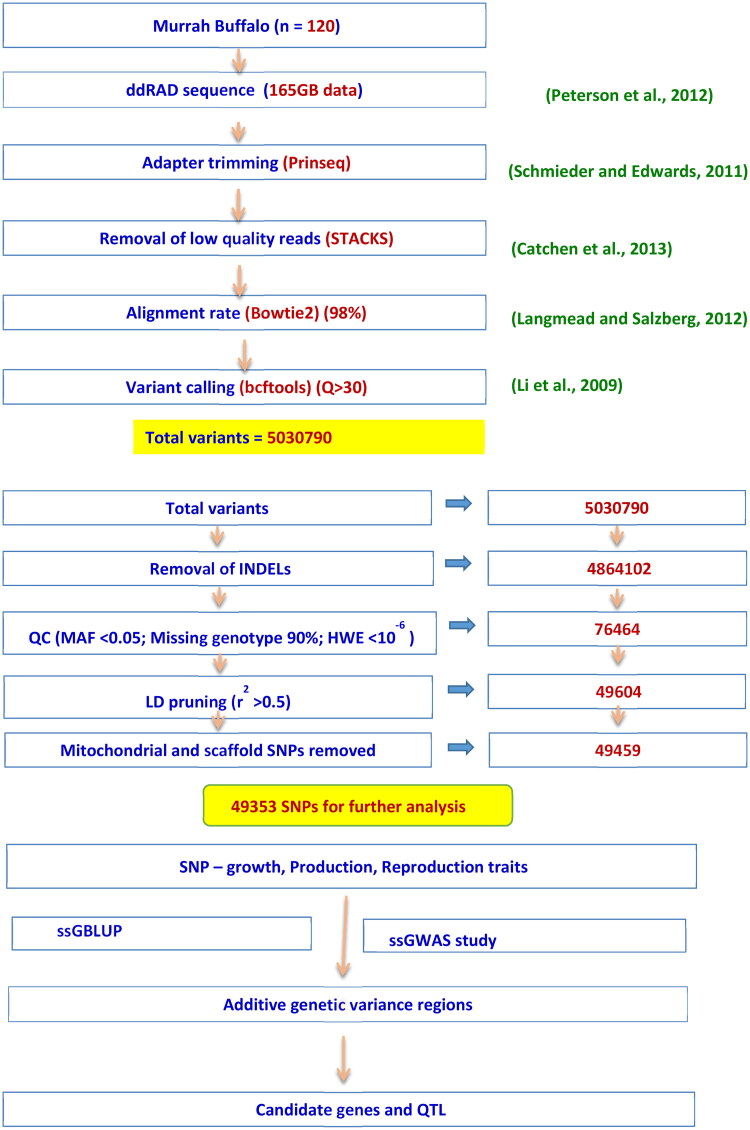
Diagrammatic representation of analysis pathway.

A total of 120 Murrah female buffaloes, which were randomly selected from the herd, were genotyped using the ddRAD method. In this study, only those buffaloes with known pedigree were considered. The quality control criteria were performed by using the PREGSF90 package under Blupf90 software^10^ that considered MAF <0.05, removing markers with call rate less than 90%, parent progeny conflicts, by which 8 animals were removed to reduce the missing heritability and deviation from HWE <10^−6^. After LD pruning, only considered autosomal and sex chromosomal SNPs for further analysis (using VCF tools excluded scaffold and mitochondrial SNPs). The missing genotypes were imputed in the BEAGLE software.^11^ Finally, 49353 SNP markers from 112 animals were available for analysis. Analysis pathway was diagrammatically presented in Figure 1.

### Phenotype data

The dataset contained information from Murrah buffaloes at the National Dairy Research Institute farm in Haryana, India. The data were available from 1965 to 2021 and comprised of 2000 animals. The pedigree contained information of 3000 animals distributed over 8 generations.

Growth traits such as weight at 6 months (6M), 12M, 18M and 24M; production traits viz. 305 day milk yield (305D MY), lactation length (LL), Dry period (DP) and reproduction traits viz. Age at First Calving (AFC), First service Period (FSP) and calving interval (CI) were considered for analysis. The descriptive statistics and genetic parameters of studied traits are presented in Table 1.

**Table 1.** Descriptive statistics and genetic parameter estimates for economically important traits in Murrah buffaloes.

Traits	Mean ± SD	*N*	h^2^±SE	Additive Var	Residual Var
6M (kg)	110.45 ± 24.33	1725	0.31 ± 0.05	145.49	322.88
12M (kg)	194.51 ± 48.26	1995	0.20 ± 0.04	416.81	1648.2
18M (kg)	268.86 ± 56.81	1951	0.30 ± 0.04	820.66	1839.4
24M (kg)	342.84 ± 64.15	2041	0.26 ± 0.04	817.9	2230.7
305D MY (kg)	1850 ± 589.62	1940	0.21 ± 0.05	66790	242530
LL (days)	315.87 ± 82.47	1943	0.08 ± 0.04	556.12	6074.7
TMY (kg)	1993.7 ± 723.6	1943	0.19 ± 0.04	92648	382950
DP (days)	173.10 ± 91.65	1327	0.22 ± 0.06	1750.8	5952.7
AFC (days)	1337.1 ± 194.4	1920	0.11 ± 0.04	4006.2	32228
CI (days)	496.37 ± 132.4	1452	0.04 ± 0.05	720.52	15553
FSP (days)	189.48 ± 131.9	1457	0.09 ± 0.05	1476.1	14558

Additive variance present in the population.

Cleaning the data set consists of removing animals with both parents missing, animals with pedigree conflicts, production below 500 kg and less than 100 days in production. Data were pruned by removing the extreme outliers (±3 SD). The fixed effects used in the analysis were specific to each trait. Fixed effects like period of birth (POB) (*n* = 9) and season of birth (SOB) (*n* = 4) were considered for growth and age at first calving traits, for production and remaining reproduction traits fixed effects like period of calving (POC) (*n* = 9), season of calving (SOC) (*n* = 4), and age at first calving (AFC) (*n* = 9) were considered, with grouping of AFC based on the Sturges formula (Sturges, 1926).

### Estimation of variance components

The variance components and genetic parameter estimates were obtained through single trait animal model using AIREML method in AIREMLF90 software.^10^^,^^12^ We used a single-step mixed linear model for the analysis. The statistical model was:

Y=Xb+Zu+e

In model additive genetic effect and residual effects were considered as random effects and POB, SOB as fixed effects for 6M, 12M, 18M, 24M body weight and AFC. POC, SOC and AFC were considered as covariables for 305D milk yield, DP, LL, FSP and CI. Where **Y** is the vector of observations, **b** is the vector of fixed effects such as POC, SOC, AFC, POB and SOB based on specific trait, **u** is the vector of direct additive genetic effects of individual animals, assumed to be normally distributed with **u** ∼ N(0, **Hσ^2^_u_**). The **H** matrix includes both non-genotyped and genotyped individuals^10^^,^^13^ **e** is the vector of random residual errors **e** ∼ N(0, **Iσ^2^_e_**). **X** and **Z** are incidence matrix related to fixed and random effects, respectively. **I** is an identity matrix, and σ^2^_u_ and σ^2^_e_ are additive genetic and residual variances, respectively.

In the mixed model equation, genetic relationship is defined using **H^−1^**, which is obtained as

$$H^{-1}=A^{-1}+[\begin{array}{cc} 0 & 0\\ 0 & G^{-1}-{A_{22}}^{-1} \end{array}]$$

where, **A^−1^** is numerator relationship matrix for all animals in the pedigree. **A_22_^−1^** is numerator relationship matrix for genotyped animals, **A_22_^−1^** is subtracted to avoid double counting of pedigree information for genotyped animals and **G^−1^** is genomic relationship matrix which was calculated as described by VanRaden et al.^6^

$$G=\frac{\text{ZDZ}\prime }{2\sum_{n\;}^{t}p_{n}q_{n}}$$

**Z** is the coefficient matrix adjusted for allele frequency, **D** is diagonal matrix of weights, **p_n_** and **q_n_** are gene frequencies and **t** is the number of SNPs. Initially **D = I**, and **I** is the identity matrix, which consider each SNP has equal contribution to phenotype and matrix dimension equal to number of genotyped animals.

Tuning of **H^−1^** matrix by giving different weights to create **G^−1^ – A_22_^−1^** to scale the genomic information to be compatible with pedigree information was done as **G^−1^** being α**G** + (1-α)**A_22_**, We have tried several values of α (0.95, 0.80 and 0.50), which is used to solve singularity issues (i.e., G^−1^ being non-positive definite). Lower values for α can hasten convergence, while having little or no effect on accuracy, and finally settled with 0.95, as it gave better accuracy than other alpha values and predictive ability. **A^−1^** was correctly constructed taking inbreeding into account^14^ while construction of **H^−1^**. In this population estimate based on pedigree inbreeding coefficient was 0.000187.

### ssGWAS using ssGBLUP

ssGWAS method was applied to estimate the marker effects using the single-step BLUP approach.^4^ According to it,

a=ZU

Where **a** is vector of breeding value for genotyped animals and **U** is vector for SNP marker effects,

$$\text{Var}\;\left(a\right)=\text{Var}\;\left(\text{ZU}\right)=G\;\sigma^{2}a=\text{ZDZ}’\sigma^{2}u$$

**D** is a diagonal matrix of weights accounting for variance of SNP markers

G=ZDZ’λ

The prediction of SNP effects $\hat{U}$ was calculated with the best predicted GEBV from blupf90 by POSTGSF90 program.

U^=Cov (U,a’)[Var(a)]−1 a^=λDZ’G−1 a^=DZ’(ZDZ’)−1 a^

After prediction of an SNP effect, we have given weights to markers based on SNP solution. The weights of SNPs were included in all analyses by iteratively applying the single-step GBLUP methodology and repeated twice, so that the effect of the SNP and the effect of the animals were recalculated in order to increase the weight of SNPs with large effects and to reduce the weight of those with small effects.

$$G(t+1)=\frac{\text{ZD}(t+1)Z\prime }{2\sum_{n\;}^{t}p_{n}q_{n}}$$

From POSTGSF90, we got results based on P value and based on proportion of additive genetic variance explained by each window of 30 adjacent SNPs. The percentage of genetic variance explained by a window segment of 30 adjacent SNPs was calculated as:

$$=\frac{\text{var}\left(a_{i}\right)}{\sigma_{a}^{2}}100$$

where, **a_i_** is the genetic value of the i^th^ region, that consists of 30 adjacent SNP, and **σ^2^_a_** is total genetic variance.

Top windows that explain more than 0.5% of additive genetic variance were used to define the important genomic regions associated with each trait. The threshold of 0.5% was chosen based on the previous reports.^15^^,^^16^ GWAS results were depicted in Manhattan plot using ‘qqman’ package of R software. NCBI genome data viewer (https://www.ncbi.nlm.nih.gov/genome/gdv) was used to identify the specific candidate genes present on the significant window region using the latest annotated UOA_WB1 buffalo reference genome. Previously reported QTL for each trait at a particular region were retrieved from animal QTLdb (https://www.animalgenome.org/cgi-bin/QTLdb/BT).

## Results

### Estimation of genetic parameters

Estimates of (Co)variance components have been derived, to get an understanding of the genetic architecture of the trait and to plan a future breeding program with a suitable direction. In Murrah buffaloes, growth, production and reproduction traits are equally important, owing to suitability of these animals for milk and meat purpose. The variance is one of the significant estimates, which is specific to the population and will determine the response to selection. The additive genetic variance present in the population was presented in Table 1. We used a single-step model to estimate the genetic parameters in the Murrah buffaloes.

The heritability estimates for growth traits were moderate to high, viz. 6M (0.31), 12M (0.20), 18M (0.30) and 24M (0.26). Our study provides the first report of genetic parameter estimates using a single-step approach in the Murrah buffaloes (Table 1). When compared with growth traits, the h^2^ estimates for production traits were sizable, viz. 305D MY (0.21), TMY (0.19), DP (0.22) and LL (0.083), Except LL, moderate h^2^ of above traits indicates that directional selection is feasible to improve these traits. Reproduction traits in our study were having lower estimates of heritability, viz. AFC (0.11), CI (0.04) and FSP (0.09).

### Wss-GWAS analysis on various economic traits

The most relevant non overlapping genotype segment (a window of 30 adjacent SNPs) explaining more than 0.5% of additive genetic variance, chromosome positions and genes for growth, production and reproduction traits were depicted in supplementary tables 1, 2 and 3, respectively. Supplementary Material Figures S1 to S10 showed the Manhattan plots with the variances of SNP windows that explain >0.5% of the additive genetic variance for various growth traits, production traits and reproduction traits, after two iterations. Details of related QTL are given in supplementary tables 4, 5 and 6 for growth, production and reproduction traits, respectively. Genomic regions in BBU (Bubalus Bubalis) chromosome 4, 6 and 15 showed an association between 6M, 12M, 18M, and 24M body weight traits. In 18M body weight trait, 7% of additive genetic variance explained by genomic region on BBU2 and the genomic region on BBU 4 and 6 explained 2% of variance, each. A total of 307 genes were identified within the genomic regions explaining more than 0.5% of the additive direct genetic variance for production traits. Genomic region of BBU4 showed an association between LL and 305D MY. In the analysis of 305D MY, genomic region on BBU9 showed maximum additive variance of 3.5% and BBU8 showed 2.7% variance. Genomic regions associated with dry period located on BBU 18 contained large number of genes with variance above 2%. Genome regions for CI showed only less additive variance when compared with AFC and FSP. For CI top genomic region explained only 0.70% variance. In FSP trait, ssGWAS analysis, genomic region on BBU 13 and 9 have shown maximum genetic variance of above 1.5%. All traits showed at least one significant region that overlapped with previously reported QTL in cattle and buffalo for growth, milk yield and reproduction traits, highlighting the pleiotropic effects of genes that cause the genetic variance which is observed. Table 2 details genomic regions and important chromosomes associated with different traits.

**Table 2.** Details of genomic regions and chromosomes associated with different traits.

Trait	No. of genomic regions	Chromosome identified with important SNP windows
**Growth traits**		
For 6M body weight	31	BBU 9, 5, 7, 24 and 23
12M body weight	19	BBU 1
18M body weight	19	BBU 2, 4, 6 and 25
24M body weight	36	BBU 11
**Production traits**		
305 day milk yield	17	BBU 9, 8, 20, 21, and 16
Lactation length	10	BBU 3 and 4
Dry period	8	BBU 18, 5, 20 and 6
**Reproduction traits**		
Age at First Calving	16	BBU 14 and 3
Calving Interval	8	BBU17
First Service Period	18	BBU 13 and 9

For body weights at different months, common regions shared were found on chromosome no. 4, in which OSBPL8, BBS10, NAP1L1, PHLDA1 genes were present. Another common region, shared by 6M and 24M BW contained MAT1A, DYDC1, DYDC2, FAM213A, TSPAN14, SH2D4B genes. These genes have lipid homeostasis regulation functions, and they generally have cell proliferation and tissue texture modeling functions. On chromosome 6, a common region shared by different growth traits, include genes PIGK, ST6GALNAC5, ST6GALNAC3, ETV3L, ARHGEF11, LRRC71, PEAR1, NTRK1, INSRR. Genomic regions associated with 6M body weight located on BBU 9 (TRNAK-UUU) and BBU5 (ZNF317) showed maximum additive variance of above 2%.

Common regions are shared by 305D MY and TMY on chromosome 8, in which the CNTNAP2 gene is present, and on chromosome 20, TGM5, MIS18BP1, FANCM, FKBP3, PRPF39, KLHL28, and FSCB were the common genes. Both traits share the PNPLA4 gene on the sex chromosome and the 4th chromosome contains the PARPBP, PMCH, NUP37, WASHC3, DRAM1, GNPTAB, SYCP3, CHPT1, MYBPC1, SPIC, ARL1, UTP20, SLC5A8, and ANO4 genes. Common chromosome regions shared by FSP and CI on chromosome 9 in which the SLC36A2 gene is present, and on chromosome 1 in which SPATA16, ECT2, NCEH1, TNFSF10, GHSR, FNDC3B, TMEM212, PLD1, TNIK genes are present.

## Discussion

Estimates of h^2^ for 6M BW ranging from 0.12 to 0.34 were reported in Murrah buffaloes in literature using a pedigree-based model.^17^^,^^18^ For 12M BW, similar results were reported.^19^^,^^20^ In congruence with our study, similar h^2^ estimate for 18M and 24M BW of 0.25 was reported,^18^^,^^21^ in Murrah buffalo. h^2^ estimates for production traits like 305D MY reported using pedigree-based models, which were in the range of 0.25 to 0.51^22–25^ instead of the reported low h^2^ (0.15) for 305DMY. For LL, literature reported low to moderate h^2^ viz. 0.10 reported in Murrah breed,^26^ 0.10 in Jaffrabadi breed.^27^ For DP, the current estimate of 0.22 was moderate, whereas literature reported low h^2^ of 0.13 in Murrah breeds.^28^

For AFC, using pedigree-based model, similar estimates were reported.^18^^,^^29^^,^^30^ For CI similar heritability were reported.^29–32^ Breed differences exist in heritability estimates, but the method of estimation used in the literature was primarily pedigree-based, either using a half-sib sire model or an animal model. The advantage of the genomic information is that the relationships between relatives are described more precisely than the pedigree-based information.^6^ The single-step model was better and resulted in sizable estimates as it considered both genotype and pedigree-based information together in one model. Sizable heritability for these traits augurs the scope for further selection in Murrah buffaloes.

Genes present on different genomic regions with high additive genetic variance have significant effect in growth. The molecular basis of RRN3 present on BBU 24 identified at 6 month body weight, regulated Pol I (Polymerase I) initiation and cell growth.^33^ It has significant regulatory effect in growth, development of tissues and organs in mammals and also for maintaining normal body weight and reproduction.^34–36^ In several cattle breeds, the TRPA1 gene (Transient receptorn potential cation channel, subfamily A, member 1) has been linked to traits such as bovine body height, body length, hucklebone width, cross ministry height, and chest width.^36^ EYA1 gene co-regulate with other genes energy balance especially in puberty of cattle.^37^ HMGN proteins have role in cell differentiation^38^ involved in early stage embryogenesis.

For 12M Body weight, the window that explained the highest percentage of additive genetic variance contained genes which were already described in previous studies for other traits. Growth associated protein (GAP43), which is found on BBU1, has been connected to the control of axonal growth and plasticity, presynaptic vesicular function, and a variety of other presynaptic proteins.^39^ NAP1L1 participates in DNA replication and modulating chromatin formation and to the regulation of cell proliferation. CAPS2 gene on BTA5 near to rs-29483 SNP was found to be associated with weaning weight in Korean native cattle.^40^ The KDM8 gene has been discovered as a potential candidate gene for daily gain and dry matter intake.^41^ KDM8 and UCK1 gene function as upstream regulators on the flanking region of SNP associated for lactation persistency in Holstein cattle.^42^ Two SNPs on BTA3 (Bovine HD0300000940 and BovineHD0300000941) within TMCO1 gene were associated with growth curve parameters, affected muscle growth and development because of significant relationship with PRKAG3 identified in Simmental beef cattle.^42^ The ALDH9A1 (aldehyde dehydrogenase 9 family, member A1), has a role on fetal growth in adult adipose tissue mass in bovine^43^ and also were associated with rib eye area in Nellore beef cattle.^44^ TDRD3 gene (BTA 12) was associated with the monounsaturated fatty acid profile in the *Longissimus thoracis* of Nellore cattle.^45^

Candidate genes present on these significant regions identified for 18 M body weight like BMP6 gene have effect on proliferation and differentiation of bone and cartilage cells and advances the production of chondrocytes and glycoproteins particular to articular cartilage.^46^ GRIA3 and THOC2 genes have effect on longevity traits in Holstein cattle.^47^

In 24M body weight trait, candidate genes present on different significant regions have already been reported in various studies. ADCK1 is critical for maintaining mitochondrial structures and functions in the muscle.^48^ LMOD3 (leiomodin-3) expressed in skeletal and cardiac muscle and also expressed from early stages of muscle differentiation.^49^ ARS-BFGL-NGS-110665 SNP present on BTA22 was associated with carcass weight and is present on FAM19A1 gene^50^ that plays vital physiological roles in neuron development and brain function.^51^

The candidate genes for 305D MY present on top chromosomes and their functions in different species have already been reported in various studies. PCSK1 gene present on BBU9 have been associated with growth traits, body weight and fat deposition in bovines.^52^ CNTNAP2 gene present in region under selection for Murrah buffaloes identified through XP-EHH and FST methodology,^53^ has important role in milk synthesis pathway in water buffalo^54^ and also associated with immunity, growth traits and calving performance in cattle.^55^

On analysis of lactation length, contain several candidate genes present at significant genomic regions explaining higher additive genetic variance. TNPO1 gene in BTA20 have role in regulation of the stature in cattle and associated with birth weight, mature weight, and yearling weight in cattle.^56^^,^^57^ In 88.07–89.60 Mb region of BTA6 with *SLC4A4, GC, NPFFR2*, and *ADAMTS3* genes have role in milk and protein yields and SCS^58^ and ADAMTS3 was associated with longevity.^59^ NPFFR2 and SLC4A4 genes present within 1 Mb window region of rs110775601 SNP associated with milk yield in Holstein cattle.^60^ GC and NPFFR2 gene reported as candidate gene for resistance to mastitis in cattle.^61^ MOB1B, DCK, SLC4A4 genes on BTA 6 were associated with milk yield and protein percentage.^62^^,^^63^

Important genes present on genomic region associated with dry period include SLC7A5 (l-type amino acid transporter 1) also known as LAT1, expressed in lactating mammary tissues of dairy cows^64^^,^^65^ increasing amino acid availability and milk protein synthesis in the mammary gland.^66^ Ca5a gene present on BTA18 was associated with productivity traits in Iraqi cattle.^67^ Zinc finger protein (*ZNF469*), CTU2, PIEZO1, CDT1, APRT, CALNS associated with milk production, mastitis resistance, immune response and heat tolerance in Sahiwal cattle present in the intergenic region of BTA 18.^68^

On analysis of AFC, different candidate genes present on significant genomic regions were explained. SNX5 as a candidate protein for the discrimination of Non pregnant heifers from pregnant ones on day 22 and high level of SNX5 in serum on day 22 can be used as a pregnancy test using PAG antibody.^69^ MGME1, OVOL2 and DZANK1 genes on BTA13 were related with scrotal circumference in Nellore cattle.^70^ Membrane associated ring-CH-type finger 11 (MARCH11) on BTA20 (rs 41956232) SNP was associated with conception rate at 1^st^ service and to repeated AI services.^71^ In pig FBXL7 was associated with different parity litter traits and it has important effect in embryonic development progression^72^ and in cattle during pregnancy increased expression of FBXL7 in endometrium.^73^ MAEL gene have vital role during spermatogenesis by repressing transposable elements, which is essential for the germline integrity.^74^ ILDR2, TADA1 and POGK genes with SNP markers on BTA 3 (2.11–2.12 Mb) were associated with age at first calving^75^ and rs41625668 SNP with conception rate.^76^

Candidate genes present in associated region of calving interval which had relationship with different traits as per literature. TRPC3 played important roles in various cells^77^ and also expressed in bovine uterine epithelium and oviducts have important role in early reproductive function.^78^ Fibroblast growth factor 2 **(**FGF2**)** on BTA6, expressed throughout in estrus cycle especially in bovine endometrium, in theca cells^79^ and in early pregnancy helps in maternal recognition of pregnancy and early embryo development.^80^^,^^81^ ARS-USMARC-528 SNP present on BTA17 (34.8 Mbp) within SPYR1 (Sprouty RTK Signaling Antagonist 1) gene was associated with gestation length.^82^

Important genes or SNP associated with service period like ATOX1, SPARC and FAT2 genes in BTA9 have significant SNP associated with heifer fertility and reproduction traits in Holstein cattle.^83^

The strength of ssGWAS is its ability to combine information from all genotypes, observed phenotypes, and pedigree information into a single simple and one-step model. A key characteristic of GWAS methods is fitting each marker one at a time as fixed effects, resulting in a lack of power to map loci for quantitative traits.^4^ This limitation has been overcome by methods such as GBLUP, which uses mixed linear models to fit high-density genome-wide molecular markers simultaneously. The GBLUP methods were extended to include information from non-genotyped individuals in prediction analysis, resulting in ssGBLUP.^10^ Putative candidate genes identified through ss-GWAS may have an influence on phenotype or inheritance. Many studies have reported other candidate genes and genomic regions associated with the traits, it may due to difference in genetic constitution, SNP covered in different sequencing methods and linkage disequilibrium.

## Conclusion

We recommend weighted single step GWAS for routine estimation of covariance components and identification significant SNPs with high genetic variance in the Murrah herd. We could observe the identified genomic regions for each trait and their effects in related traits. An important part of genetic variance of different economic traits was explained by different genes in each chromosome identified through WssGWAS in Murrah buffalo. These results can be used to find the causative mutations with high additive variance for marker assisted selection to improve the economic traits. However, the genomic data used in this study was of low magnitude, and hence we recommend further studies using large number of animals genotyped. The utility of whole genome sequenced data in future will help to validate the association and identify the causal mutation.

## Supplementary Material

Supplemental Material
