# Supplementary material for: Weighted single step GWAS reveals genomic regions associated with economic traits in Murrah buffaloes
Source: Anim Biotechnol. 2024 Mar 4;35(1):2319622. doi: 10.1080/10495398.2024.2319622 (PMC12674339; doi:10.1080/10495398.2024.2319622)
Supplement: Supplemental Material [file LABT_A_2319622_SM3923.zip › final_growth.docx]

**Supplementary Table 1. Genes for growth traits in Murrah buffaloes, their positions in base pairs (bp) and descriptions based on windows of 30 adjacent SNP explaining at least 0.5% of the trait genetic variance**

| Trait | chromosome | Variance | Start position | End position | Genes |
| --- | --- | --- | --- | --- | --- |
| 6M body weight | 9 | 2.89714 | 97478176 | 98000849 | TRNAK-UUU |
|  | 5 | 2.09167 | 70321188 | 72377066 | ZNF317 |
|  | 7 | 1.90333 | 11413980 | 11926642 | TRNAA-UGC, CLNK |
|  | 24 | 1.58167 | 28255245 | 28995510 | BMERB1, MPV17L, PDXDC1, NTAN1, RRN3, BFAR, PARN |
|  | 23 | 1.51018 | 11587721 | 12640777 | PANK1, KIF20B, TRNAC-GCA |
|  | 15 | 1.29087 | 46529806 | 47306539 | TRPA1, TRNAE-UUC, MSC, EYA1 |
|  | 1 | 1.27953 | 184060800 | 184833488 | TRNAC-GCA, PSMG1, BRWD1, HMGN1, GET1, LCA5L, SH3BGR, B3GALT5, IGSF5 |
|  | 3 | 1.18154 | 122995495 | 124814207 | PAX5, ZCCHC7, GRHPR, ZBTB5, POLR1E, FBXO10, TOMM5, FRMPD1, TRNAC-GCA, TRMT10B, EXOSC3, DCAF10, EXOSC3, DCAF10, SLC25A51, SHB, TRNAC-GCA, RECK, GLIPR2, CCIN, CLTA, GNE, RNF38, MELK |
|  | 21 | 1.09837 | 55123954 | 56948966 | HRH1, ATG7, VGLL4, TAMM41, TRH, TMCC1, PLXND1, RHO, IFT122, MBD4, EFCAB12, RPL32, CAND2, TMEM40, RAF1, NNKRN2, MKRN2OS |
|  | 4 | 1.07055 | 113284866 | 114785499 | E2F7, CSRP2, ZDHHC17, OSBPL8, BBS10, NAP1L1, PHLDA1 |
|  | 19 | 1.05158 | 42349981 | 45674231 | TRNAY-GUA, TRNAC-GCA |
|  | 7 | 1.01288 | 23848641 | 24953011 | PAQR3, BMP2K, TRNAC-GCA, ANXA3, FFRAS1, MRPL1 |
|  | 6 | 0.98684 | 11118513 | 14056304 | SPTA1, KIRREL1, TRNAH-AUG, CD5L, FCRL1, FCRL3, FCRL5, FCRL4, TRNAC-ACA, ETV3L, ARHGEF11, LRRC71, PEAR1, NTRK1, INSRR |
|  | 3 | 0.9596 | 17495946 | 18685912 | NSF, WNT3, WNT9B, GOSR2, RPRML, LYZL6, RDM1, PLEKHM1, ARHGAP27, MAP3K14, SPATA32,, FMNL1, HEXIM2, HEXIM1, ACBD4, PLCD3, NMT1, DCAKD, C1QL1, KIF18B, FAM187A. CCDC103, EFTUD2, HIGD1B, GJC1, ADAM11, DBF4B, CCDC43, MEIOC |
|  | 8 | 0.95003 | 22142374 | 24728833 | DGKB, AGMO, MEOX2, CRPPA, SOSTDC1, LRRC72. |
|  | 17 | 0.886 | 67191589 | 68439394 | FBXW7, TRNAY-GUA, TMEM154, TIGD4, ARFIP1, FHDC1, TRIM2, MND1, TMEM131L, TLR2, RNF175 |
|  | 11 | 0.8734 | 95587196 | 96596882 | POC5, ANKDD1B, POLK, CERT1, HMGCR, TRNAY-GUA, ANKRD31, GCNT4 |
|  | 4 | 0.83679 | 158427671 | 159900282 | CCSER2, TRNAR-GCG, GRID1 |
|  | 3 | 0.83537 | 48460470 | 49419957 | AP2B1, RASL10B, GAS2L2, MMP28, TAF15, HEATR9, CCL5, CCL16, CCL3, HEATR6, HNF1B, DDX52, SYNRG, DUSP14 |
|  | 8 | 0.82801 | 49526994 | 51426366 | STARD3NL, CAPZA2, CTTNBP2, CFTR, ASZ1, WNT2, ST7, MET |
|  | 1 | 0.78056 | 25290611 | 26815790 | TRNAW-CCA, FGF20, MICU3, ZDHHC2, CNOT7, VPS37A, MTMR7, TRNAE-UUC, SLC7A2, PGDFRL, MTUSC1, FGL1, PCM1, ASAH1, FRG1 |
|  | 5 | 0.76904 | 40628939 | 42739999 | FASLG, SUCO, PIGC, DNM3, METTL13, VAMP4, MYOC, PRRC2C, FMO4,, FMO1, FMO2, MROH9 |
|  | 8 | 0.64675 | 114290122 | 115427884 | KMT2C, CCT8L2, XRCC2, ACTR3B, TRNAY-AUA, DPP6 |
|  | 3 | 0.62677 | 28009274 | 30113903 | TNFRSF12B, PLD6, FLCN, COPS3, NT5M, MED9, RASD1, PEMT, RAI1, SREBF1, TOM1L2, DRC3, ATPAF2, GID4, DRG2, MYO154A, ALKBH5, LLGL1, FL11, MIEF2, TOP3A, SMCR8, SHMT1, PRPSAP2, SLC5A10, FAM83G, EPN2, B9D1, MAPK7, MFAP4, RNF112, TRNAW-CCA, SLC47A1, ALDH3A2, SLC47A2, ALDH3A1, ULK2, AKAP10, SPECC1, ADORA2B, TTC19, NCOR1, PIGL, CENPV, UBB, TRPV2, LRRC75A, ZNF287, ZNF624, ZNF286A, TRIM16, CDRT1 |
|  | 1 | 0.61353 | 102164971 | 104773640 | ABHD10, TAGLN3, TMPRSS7, GCSAM, SLC9C1, CD200, BTLA, ATG3, SLC35A5, CCDC80, GTPBP8, NEPRO, BOC, CFAP44, SPICE1, SIDT1, USF3, NAA50, ATP6V1A, GRAMD1C, ZDHHC23, CCDC191, QTRT2, DRD3, TIGIT, ZBTB20 |
|  | 19 | 0.61271 | 59292613 | 60977633 | DNAH5 |
|  | 6 | 0.59087 | 108224413 | 108756789 | GRIK3 |
|  | 9 | 0.56993 | 92080889 | 93735083 | KDM4B, PTPRS, ZNRF4, SAFB2, SAFB, TRNAK-UUU, MICOS13, HSD11B1L, RPL36, LONP1, CATSPERD, PRR22, DUS3L, NRTN, NDUFA11, VMAC, CAPS, RANBP3, RFX2, ACSBG2, MLLT1 |
|  | 4 | 0.54669 | 154796749 | 155728398 | MAT1A, DYDC1, DYDC2, PRXL2A, TSPAN14, SH2D4B, TRNAW-CCA |
|  | 1 | 0.53407 | 176928549 | 178652501 | IL2ORB, NCK1, SLC35G2, STAG1, TRNAF-AAA, PCCB, TRNAG-CCC, MSL2, PPP2R3A, TRNAR-GCG |
|  | 21 | 0.52306 | 35744327 | 37547672 | MAGI1, ADAMTS9, PRICKLE2, TRNAC-GCA, PSMD6, ATXN7 |
| 12M body weight | 1 | 1.70456 | 104212280 | 106677614 | GAP43,LSAMP, TRNAK-UUU, ZBTB20 |
|  | 4 | 1.05671 | 113991419 | 115949196 | OSBPL8,BBS10, NAP1L1,PHLDA1,KRR1, GLIPR1, GLIPR1L2, GLIPR1L1,CAPS2, KCNC2, TRNAY-GUA |
|  | 24 | 0.99382 | 17667918 | 18735904 | IL4R,NSMCE1, KDM8, TRNAD-AUC |
|  | 6 | 0.8707 | 2470057 | 3107024 | FAM78B, UCK1, TMCO1, ALDH9A1, MGST3 |
|  | 15 | 0.81484 | 68531163 | 69597491 | FAM84B |
|  | 13 | 0.80415 | 87871869 | 89656136 | DIAPH3, TDRD3 |
|  | 10 | 0.76931 | 75836030 | 77154266 | FABP7, SMPDL3A, CLVS2, TRDN, NKAIN2 |
|  | 15 | 0.70738 | 16840483 | 18287550 | OSR2, VPS13B, RGS22, FBX043, POLR2K, SPAG1, RNF19A |
|  | 4 | 0.64677 | 96639724 | 97548558 | CRADD, SOCS2, MRPL42, UBE2N, NUDT4, EEA1 |
|  | 2 | 0.61457 | 144968021 | 147293845 | ICOS, TRNAS-GGA, PARD3B, NRP2, INO80D, NDUFS1 |
|  | 7 | 0.58574 | 57117325 | 58712502 | LIMCH1, UCHL1, APBB2, NSUN7, RBM47, TRNAG-CCC, CHRNA9, TRNAG-GCC, RHOH, PDS5A, UBE2K |
|  | 5 | 0.57214 | 18114025 | 19663684 | CACNA1E, IER5, STX6, KIAA1614, XPR1, ACBD6, LHX4, QSOX1 |
|  | 15 | 0.56946 | 27780184 | 30549429 | CSMD3 |
|  | 6 | 0.55994 | 66561616 | 67876226 | AK5, PIGK, ST6GALNAC5, ST6GALNAC3 |
|  | 16 | 0.5332 | 12630834 | 13625631 | LRRC4C |
|  | 4 | 0.52451 | 85338234 | 87196049 | ANO6,ARID2, TRNAC-ACA, SCAF11, SLC38A1, SLC38A2, TRNAC-GCA, SLC38A4, PCED1B,AMIGO2 |
|  | 17 | 0.52202 | 31712751 | 32971180 | FAM198B, TMEM144, RXFP1, ETFDH, PPID, FNIP2 |
|  | 25 | 0.51971 | 37988461 | 39900822 | BCOK, ATP6AP2, MPC1L, MED14, MED14OS, TRNAE-UUC |
|  | 7 | 0.51076 | 44665591 | 46594109 | IGFBP7, POLR2B, REST, HOPX, THEGL, ARL9, SRP72, PPAT, AASDH, KIAA1211, CEP135, EXOC1,EXOC1L, NMU, PDCL2, CLOCK, TMEM165 |
| 18M body weight | 2 | 7.5785 | 4176814 | 5495798 | RREB1, SSR1, CAGE1, DSP, SNRNP48, BMP6, TXNDC5, BLOC1S5, EEF1E1, SLC35B3 |
|  | 4 | 2.91377 | 114311624 | 116077937 | BBS10, NAP1L1, PHLDA1, KRR1, GLIPR1, GLIPR1L2, GLIPR1L1, CAPS2, KCNC2, ATXN7L3B |
|  | 6 | 2.12176 | 66869546 | 68176949 | PIGK, ST6GALNAC5, ST6GALNAC3 |
|  | 25 | 1.53929 | 120819690 | 122928405 | GRIA3, THOC2, XIAP, STAG2, TEX13D, SH2D1A, TENM1 |
|  | 1 | 0.99293 | 19064123 | 20268848 | DCTN6,MBOAT4, LEPROTL1, SARAF,DUSP4, TNKS |
|  | 25 | 0.85192 | 37333065 | 39534689 | SRPX, RPGR, OTC, TSPANT, MID1IP1, BCOR, ATP6AP2,MPC1C |
|  | 12 | 0.81935 | 53084298 | 54866859 | CTNNA2, CRRTM1 |
|  | 7 | 0.78833 | 61586313 | 63685212 | DTHD1, ARAP2 |
|  | 1 | 0.75685 | 39725446 | 40122790 | Loc102405229 |
|  | 16 | 0.74503 | 66566297 | 68750808 | DDX1O, ATM, ACAT1, CUL5, RAB39A, SLC35F2,SLN, ELMOD1, ALKBH8, CWF19L2 |
|  | 13 | 0.69626 | 42702470 | 45410595 | DACH1, KLF5, PIBF1, DIS3, MZT1 |
|  | 19 | 0.61138 | 51530571 | 53012548 | TRNAC-GCA |
|  | 2 | 0.58976 | 171325766 | 172067031 | CAB39, ITM2C, GPR55, SPATA3, PSMD1, HTR2B, ARMC9 |
|  | 13 | 0.57995 | 9423597 | 10304104 | NALCN, TMTC4, GGACT, PCCA |
|  | 4 | 0.55528 | 125698954 | 126176670 | MAP3K21, KCNK1 |
|  | 17 | 0.54041 | 31712751 | 32971180 | FAM198B, TMEM144, RXFP1, ETFDH, PPID, FNIP2 |
|  | 5 | 0.5314 | 110471244 | 110935037 | NTM |
|  | 15 | 0.52767 | 3011033 | 4735689 | RALYL |
|  | 11 | 0.51545 | 93073607 | 94929791 | LHFPL2, SCAMP1, AP3B1, TBCA, OTP, WDR41, PDE8B, ZBED3, AGGF1, CRHBP, S100Z, F2RL, F2R, IQGAP2, F2RL2 |
| 24M body weight | 11 | 1.73454 | 14198102 | 15307825 | ADCK1, SNW1, SLIRP, ALKBH1, SPTLC2, ISM2, AHSA1, VIPAS39, NOXRED1, SAMD15, TMED8, GSTZ1, POMT2, NGB, TMEM63C, ZDHHC22, CIPC, IRF2BPL, LRRC74A, ANGEL1, VASH1 |
|  | 18 | 1.39844 | 17251983 | 18011174 | CBLN1, ZNF423 |
|  | 21 | 1.31835 | 31962401 | 35054379 | FRMD4B, LMOD3, ARL6IP5, UBA3, TMF1, EOGT, FAM19A4, FAM19A1, SUCLG2, KBTBD8, LRIG1 |
|  | 4 | 1.07527 | 28601916 | 29489168 | PIK3C2G, PLCZ1, CAPZA3, PLEKHA5 |
|  | 22 | 1.03224 | 44476091 | 47522192 | TRNAC-GCA |
|  | 15 | 1.00013 | 18899099 | 19941891 | ZNF706, GRHL2, NCALD |
|  | 14 | 0.95666 | 5187110 | 6917367 | CEBPB, TMEM189, UBE2V1, SNAI1, RNF114, SPATA2, SLC9A8, B4GACT5, PTGIS,KCNB1, ZNFX1, DDX27, STAU1, CSE1L, ARFGEF2, PREX1 |
|  | 6 | 0.92966 | 12869155 | 14587454 | ETV3, ETV3L, ARHGEF11, LRRC71, PEAR1, NTRK1, INSRR, SH2D2A, PRCC, HDGF, MRPL24, RRNAD1, ISG20L2, CRABP2, NES, BCAN, HAPLN2, GPATCH4, NAXE, TTC24, IQGAP3, MEF2D, RHBG, TSACC, CCT3, GLMP, TMEM9, SMG5, PAQR6, BGLAP, SLC25A44, SEMA4A, LMNA, MEX3A, RAB25, LAMTOR2, UBQLN4, SSR2, ARHGEF2, RXFP4, KHDC4, RIT1, SYT11 |
|  | 2 | 0.83838 | 2727846 | 4841866 | DYL, RPP40, PPPIR3G, LYRM4, FARS2, NRN1, F13A1, LY86, RREB1, SSR1, CAGE1, RIOK1, DSP, SNRNP48, BMP6 |
|  | 4 | 0.8064 | 48194422 | 49110885 | SYN3, TIMP3, FBXO7, BPIFC, RTCB, ASCL4, PRDM4, PWP1, BTBD11 |
|  | 18 | 0.79497 | 48290507 | 49184426 | LGALS7, LGALS4, ECH1, HNRNPL, RINL, SIRT2, NFKBIB, CCER2, SARS2, MRPS2, FBXO17, ACP7, PAK4, NCCRP1, SYCN, IFNL3, LRFN1, GMFG, SAMD4B, PAF1, MED29, ZFP36, PLEKHG2, RPS16, SUPT5H, TIMM50, DLL3, SELENOV, LEUTX, DYRK1B, FBL |
|  | 16 | 0.7721 | 9206522 | 10018475 | SYT13, PRDM11, TP53I11, TSPANI8 |
|  | 17 | 0.7708 | 68153619 | 69059521 | ARFIP1, FHDC1, TRIM2, MND1, TMEM131L |
|  | 4 | 0.71096 | 72525584 | 74152621 | LLPH, TMBIM4, IRAK3, HELB, GRIP1, CAND1, DYRK2 |
|  | 5 | 0.70173 | 57085072 | 57955356 | MARC2, MAK1, RAB3GAP2, IARS2, BPNT1, EPRS, SLC30A10 |
|  | 9 | 0.67857 | 76282921 | 77221353 | DTWD2, DMXL1, TNFAIP8,HSD17B4, FAM170A |
|  | 23 | 0.67609 | 17728543 | 18868831 | PIK3AP1, LCOK, SLIT1, ARHGAP19, FRAT1, FRAT2, RRP12, PGAM1, EXOSC1, ZDHHC16, MMS19, UBTD1, ANKRD2, HOGA1, MORN4, PI4K2A, AVPI1, MARVELD1,ZFYVE27, SFRP5, GOLGA7B, CRTAC1 |
|  | 6 | 0.67561 | 96275646 | 97533853 | AGBL4, BEND5, SPATA6, SLC5A9 |
|  | 4 | 0.6599 | 154796749 | 155728398 | MAT1A, DYDC1, DYDC2, FAM213A, TSPAN14, SH2D4B |
|  | 13 | 0.65824 | 65154697 | 66357686 | DCLK1, SPART, CCNA1, SERTM1, RFXAP, SMAD9, ALG5, EXOSC8, SUPT20H |
|  | 3 | 0.6559 | 172512141 | 174120753 | CDK5RAP2, MEGGF9, FBXW2, PSMD5, PHF19, TRAF1, C5, CNTRL, RAB14, GSN, STOM, DCDC2C, ALLC, COLEC11, RPS7, RNASEH1, ADI1, TRAPPC12, EIPR1 |
|  | 2 | 0.65225 | 95778093 | 96679519 | ARL6IP6, PRPF40A, FMNL2, STAM2, CACNB4 |
|  | 21 | 0.64934 | 7775393 | 8874934 | CLASP2, PDCD6IP |
|  | 7 | 0.62255 | 98696695 | 100908760 | PPP3CA, EMCN, DDIT4L, DNAJB14, LAMTOR3, DAPP1, MTTP, TRMT10A |
|  | 5 | 0.60033 | 110471244 | 110935037 | NTM |
|  | 14 | 0.59622 | 50615340 | 53311809 | EPC1, NSUN6, CACNB2, SLC39A12, MRC1, TMEM236, STAM, HACD1, ST8SIA6, VIM, TRDMT1, CUBN, RSU1, C1QL3, PTER |
|  | 10 | 0.59469 | 6514034 | 6947382 | PRKN |
|  | 14 | 0.59038 | 65192852 | 66609301 | PARD3, CUL2, CREM, CCNY, APBB1IP, PDSS1, ABI1, ACBD5, MASTL, YME1L1, CCDC3, CAMK1D |
|  | 3 | 0.57712 | 13245423 | 15086355 | FOXK2, WDR45B,RAB40B, FN3KRP, FN3K, TBCD, ZNF750, B3GNTL1, METRNL, PSMD12,PITPNC1, NOL11, BPTF, KPNA2, SMURF2, CEP95, DDX5, POLG2, MILR1, PECAM1, TEX2, ERN1 |
|  | 5 | 0.57326 | 13245423 | 15086355 | PTGS2, PDC, ODR4, TPR, PRG4, HMCN1, IVNS1ABP, SWT1, TRMT1L, RNF2, FAM129A |
|  | 9 | 0.56794 | 89420226 | 91410597 | RAD50, IL13, IL4, KIF3A, CCNI2, SEPT8, MKNK2, MOB3A, IZUMO4, AP3D1, JSRP1, AMH, SF3A2, PLEKHJ1, DOT1L, OAZ1, PEAK3, LINGO3, LSM7, SPPL2B, TMPRSS9, TIMM13, LMNB2, GADD45B, GNG7, DIRAS1, SLC39A3, SGTA, THOP1, ZNF554, TLE6, TLE2, AES, GNA11, GNA15, S1PR4, NCLN, CELF5, NFIC, SMIM24, DOHH, FZR1, MFSD12, HMG20B, GIPC3, TBXA2R, CACTIN, PIP5K1C, TJP3, APBA3, MRPL54, RAX2, MATK, ZFR2, ATCAY, NMRK2, DAPK3, EEF2, PIAS4, ZBTB7A, MAP2K2 |
|  | 2 | 0.56162 | 175554475 | 176384398 | LAPTM5, MATN1 |
|  | 23 | 0.53139 | 42948866 | 43258591 | CPXM2, CHST15 |
|  | 3 | 0.52773 | 123438813 | 124989848 | RNF38, MELK, PAX5, ZCCHC7, GRHPR, ZBTB5, POLR1E, FBXO10, TOMM5, FRMPD1, TRMTIOB, EXOSC3, DCAF10, SHB |
|  | 5 | 0.51997 | 91447138 | 92703055 | TENM4 |
|  | 6 | 0.51207 | 729583 | 1620102 | DCAF6, MPC2, ADCY10, MPZL1, RCSD1, CREG1, CD247, POU2F1, DUSP27 |
