# Supplementary material for: Weighted single step GWAS reveals genomic regions associated with economic traits in Murrah buffaloes
Source: Anim Biotechnol. 2024 Mar 4;35(1):2319622. doi: 10.1080/10495398.2024.2319622 (PMC12674339; doi:10.1080/10495398.2024.2319622)

Supplementary figures S1 show the Manhattan plots with the variances of SNP windows of 30 SNPs that explain >0.5% of the additive genetic variance for 6M body weight after two iterations. X-axis shows chromosome wise SNPs and Y-axis shows percentage of genetic variance explained


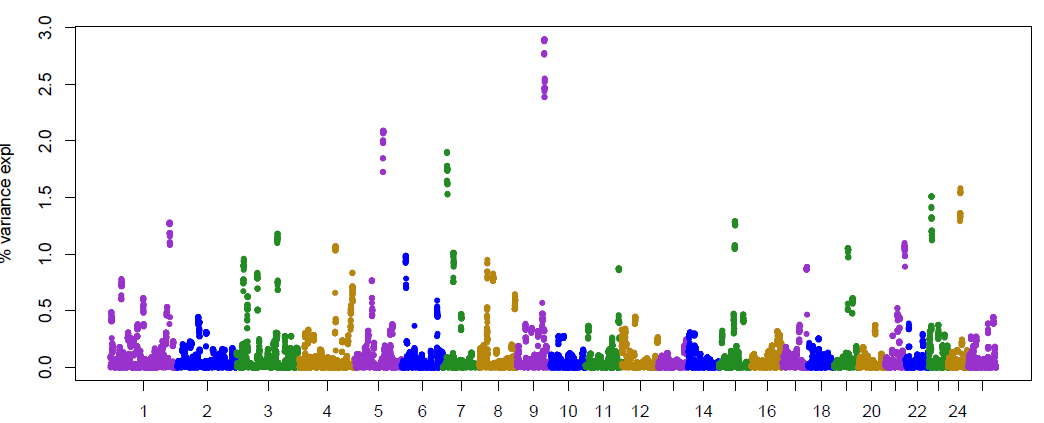


Supplementary figures S2 show the Manhattan plots with the variances of SNP windows of 30 SNPs that explain >0.5% of the additive genetic variance for 12M body weight after two iterations. X-axis shows chromosome wise SNPs and Y-axis shows percentage of genetic variance explained


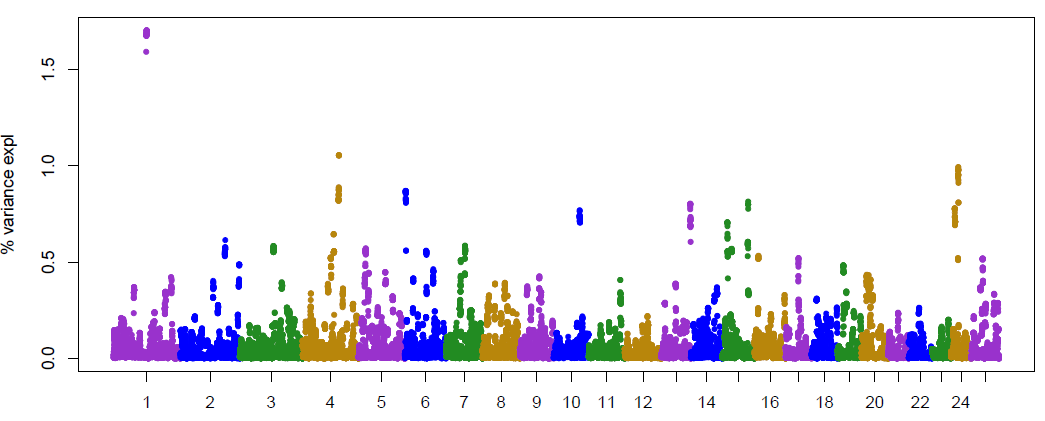


Supplementary figures S3 show the Manhattan plots with the variances of SNP windows of 30 SNPs that explain >0.5% of the additive genetic variance for 18M body weight after two iterations. X-axis shows chromosome wise SNPs and Y-axis shows percentage of genetic variance explained


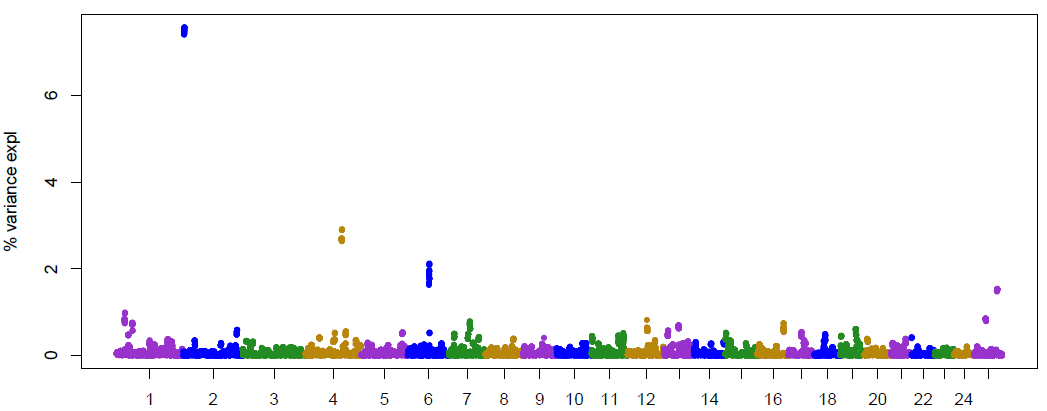


Supplementary figures S4 show the Manhattan plots with the variances of SNP windows of 30 SNPs that explain >0.5% of the additive genetic variance for 24M body weight after two iterations. X-axis shows chromosome wise SNPs and Y-axis shows percentage of genetic variance explained


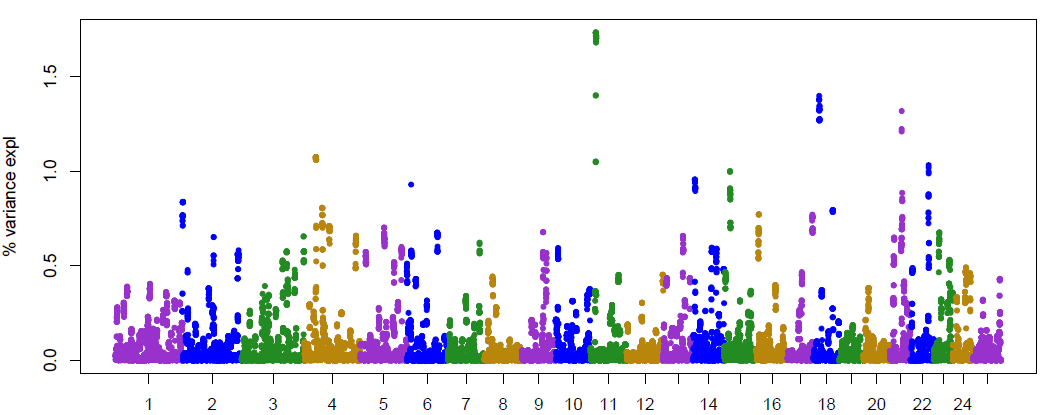

Supplement: Supplemental Material [file LABT_A_2319622_SM3923.zip › manhattan_plot_growth.docx]
