# Supplementary material for: Weighted single step GWAS reveals genomic regions associated with economic traits in Murrah buffaloes
Source: Anim Biotechnol. 2024 Mar 4;35(1):2319622. doi: 10.1080/10495398.2024.2319622 (PMC12674339; doi:10.1080/10495398.2024.2319622)
Supplement: Supplemental Material [file LABT_A_2319622_SM3923.zip › qtl_Wssgwas.docx]

**Supplementary table 4: Relevant QTLs, their positions in base pairs (bp) in cattle chromosome (chr), based on the selected region that explains 0.5% on the windows of 30 adjacent SNPs variance for the growth traits**

| Trait | chr | Var | Start position | End position | QTL position | Trait | Reference |
| --- | --- | --- | --- | --- | --- | --- | --- |
| 6 Month | 9 | 2.89714 | 97478176 | 98000849 | 9:97191669-97521014 bp | Average daily gain | Peters et al., 2012 |
|  | 5 | 2.09167 | 70321188 | 72377066 | 5:71801045-71801085 bp | Hip height | An et al., 2019 |
|  | 24 | 1.58167 | 28255245 | 28995510 | 24:28907134-28907174 bp | Body weight | Snelling et al., 2010 |
|  | 15 | 1.29087 | 46529806 | 47306539 | 15:12498658-12498698 bp | Maturity rate | Crispim et al., 2015 |
|  | 4 | 1.07055 | 113284866 | 114785499 | 4:113506072-113506112 bp  4:113614744-113614784 bp  4:113303874-113303914 bp | Average daily gain  Body weight | Akanno et al., 2018 |
|  | 7 | 1.01288 | 23848641 | 24953011 | 7:23000609-23994402 bp  7:23908372-23908412 bp | Body weight  Body weight gain | Saatchi et al., 2014  Snelling et al., 2010 |
|  | 6 | 0.98684 | 11118513 | 14056304 | 6:0-37983812 bp | Body weight | Saatchi et al., 2014 |
|  | 8 | 0.95003 | 22142374 | 24728833 | 8:23870961-23871001 bp | Body weight | Snelling et al., 2010 |
|  | 17 | 0.886 | 67191589 | 68439394 | 17:67546190-67546230 bp | Body weight gain | Snelling et al., 2010 |
|  | 11 | 0.8734 | 95587196 | 96596882 | 11:95837973-95838013 bp | Body weight gain | Snelling et al., 2010 |
|  | 3 | 0.62677 | 28009274 | 30113903 | 3:28087327-28087367 bp | Body weight gain | Snelling et al., 2010 |
|  | 1 | 0.61353 | 102164971 | 104773640 | 1:102864175-102864215 bp | Body weight gain | Snelling et al., 2010 |
|  | 6 | 0.59087 | 108224413 | 108756789 | 6:108444266-108444306 bp | Body weight gain | Snelling et al., 2010 |
|  | 9 | 0.56993 | 92080889 | 93735083 | 9:93675921-93675961 bp | Average daily gain | Seabury et al., 2017 |
|  | 21 | 0.52306 | 35744327 | 37547672 | 21:35921970-35922010 bp  21:36186083-36186123 bp | Body weight  Average daily gain | Porto-Neto et al., 2015  Akanno et al., 2018 |
| 12 Month | 1 | 1.70456 | 104212280 | 106677614 | 1: 105727470-105727510 bp | Dry matter intake and growth | Seabury et al., 2017 |
|  | 4 | 1.05671 | 113991419 | 115949196 | 4:114302462-114302502 bp  4:115170119-115170159 bp  4:115581035-115581075 bp | Body weight  Body weight gain  Carcass weight | Snelling et al., 2010  Akanno et al., 2018 |
|  | 6 | 0.8707 | 2470057 | 3107024 | 6:0-37983812 bp | Body weight | Saatchi et al., 2014 |
|  | 15 | 0.81484 | 68531163 | 69597491 | 15:69258016-69258056 bp | Maturity rate | Crispim et al., 2015 |
|  | 24 | 0.78058 | 10731584 | 11172427 | 24:11016626-11016666 bp | Body weight gain | Snelling et al., 2010 |
|  | 5 | 0.57214 | 18114025 | 19663684 | 5:18940413-19018750 bp | body weight | Zhuang et al., 2020 |
|  | 4 | 0.52451 | 85338234 | 87196049 | 4:86725330-86725370 bp | Body weight | Snelling et al., 2010 |
|  | 25 | 0.51971 | 37988461 | 39900822 | 25:38330904-38330944 bp | Body weight | Akanno et al., 2018 |
| 18 Month | 2 | 7.5785 | 4176814 | 5495798 | 2:2814353-4339368 bp | Age at puberty | Stafuzza et al., 2020 |
|  | 4 | 2.91377 | 114311624 | 116077937 | 4:115170119-115170159 bp | Body weight gain | Snelling et al., 2010 |
|  | 6 | 2.12176 | 66869546 | 68176949 | 6:66905274-66905314 bp  6:67105966-67106006 bp | Maturity rate  Body weight gain | Crispim et al., 2015  Snelling et al., 2010 |
|  | 25 | 0.85192 | 37333065 | 39534689 | 25:38330904-38330944 bp | Body weight | Snelling et al., 2010 |
|  | 12 | 0.81935 | 53084298 | 54866859 | 12:53100756-53100796 bp | Withers height | Wu et al., 2013 |
|  | 1 | 0.75685 | 39725446 | 40122790 | 1:40046159-40046199 bp | Age at puberty | Hawken et al., 2011 |
|  | 16 | 0.74503 | 66566297 | 68750808 | 16:67192672-67192712 bp | Body depth | Cole et al., 2011 |
|  | 13 | 0.69626 | 42702470 | 45410595 | 13:43113673-43113713 bp | Body weight gain | Snelling et al., 2010 |
|  | 13 | 0.57995 | 9423597 | 10304104 | 13:9970466-9970506 bp | Maturity rate | Crispim et al., 2015 |
|  | 5 | 0.5314 | 110471244 | 110935037 | 5:110704138-110704178 bp | Body weight | Snelling et al.,2010 |

| 24 Month | 11 | 1.73454 | 14198102 | 15307825 | 11:14260820-14260860 bp | Body weight | Crispim et al., 2015 |
| --- | --- | --- | --- | --- | --- | --- | --- |
|  | 18 | 1.39844 | 17251983 | 18011174 | 18:17672698-17672738 bp | Metabolic body weight | Seabury et al., 2017 |
|  | 4 | 1.07527 | 28601916 | 29489168 | 4:29193719-29193759 bp | Age at puberty | Hawken et al., 2012 |
|  | 22 | 1.03224 | 44476091 | 47522192 | 22:44505184-44505224 bp  22:45326855-45326895 bp | Average daily gain  Maturity rate | Seabury et al., 2017  Crispim et al., 2015 |
|  | 6 | 0.92966 | 12869155 | 14587454 | 6:0-37983812 bp  6:0-40983913 bp  6:13711248-13711288 bp | Body weight  Body weight  Average daily gain | Saatchi et al., 2014  Xu et al., 2019 |
|  | 2 | 0.83838 | 2727846 | 4841866 | 2:3904091-3904131 bp | Maturity rate | Crispim et al., 2015 |
|  | 4 | 0.8064 | 48194422 | 49110885 | 4:49010857-49010897 bp | Body weight gain | Snelling et al., 2010 |
|  | 18 | 0.79497 | 48290507 | 49184426 | 18:48616931-48616971 bp | Body weight gain | Snelling et al., 2010 |
|  | 16 | 0.7721 | 9206522 | 10018475 | 16:9440077-9440117 bp  16:9211957-9211997 bp  16:9464905-9464945 bp | Average daily gain  Average daily gain  Body depth | Xu et al., 2019  Rolf et al., 2012  Cole et al., 2011 |
|  | 4 | 0.71096 | 72525584 | 74152621 | 4:73894110-73894150 bp | Body weight | snelling et al., 2010 |
|  | 2 | 0.65225 | 95778093 | 96679519 | 2:95884123-95884163 bp | Body weight gain | Snelling et al., 2010 |
|  | 5 | 0.60033 | 110471244 | 110935037 | 5:110704138-110704178 bp | Body weight | Snelling et al., 2010 |
|  | 9 | 0.56794 | 89420226 | 91410597 | 9:90417787-90428353 bp | Average daily gain | Xu et al., 2019 |
|  | 6 | 0.51207 | 729583 | 1620102 | 6:0-37983812 bp | Body weight | Saatchi et al., 2019 |

**Supplementary table 5: Relevant QTLs, their positions in base pairs (bp) in cattle chromosome (chr), based on the selected region that explains 0.5% on the windows of 30 adjacent SNPs variance for the production traits**

| Traits | chro | var | start | End | QTL POSITION | trait | reference |
| --- | --- | --- | --- | --- | --- | --- | --- |
| 305 day milk yield | 9 | 3.52253 | 12192481 | 15311807 | 9:12632105-12632145 bp | Milk yield, fat and protein percentage | Cole et al., 2011 |
|  | 20 | 1.40315 | 50726448 | 52054688 | 20:50828097-50828137 bp | Milk yied, fat and protein yield | Pryce et al., 2010 |
|  | 20 | 1.37991 | 14110785 | 16626498 | 20:13731671-15651653 bp  20:14223022-14223062 bp | Milk fat yield  Milk protein percentage | Oliveira et al., 2019  Cole et al., 2011 |
|  | 21 | 1.36151 | 39019077 | 39895292 | 21:38049838-39184077 bp | Milk fat yiled | Oliveira et al., 2019 |
|  | 16 | 1.00392 | 46251522 | 47885362 | 16:47367664-47367704 bp  16:47804765-47804805 bp | Milk yield and milk protein yield  Milk fat percentage | Jiang et al., 2019  Buitenhuis et al., 2014 |
|  | 2 | 0.89372 | 31488482 | 32558439 | 2:31968871-31968911 bp | Milk kappa casein percentage | Buitenhuis et al., 2014 |
|  | 2 | 0.88508 | 89761245 | 91263644 | 2:91118611-91118651 bp | Milk protein yield | Zhou et al., 2019 |
|  | 25 | 0.84115 | 4790673 | 5254724 | 25:4794624-4794664 bp  25:4902427-4902467 bp | Milk fat yield | Iso-Touru et al., 2016 |
|  | 13 | 0.79673 | 59545493 | 60180470 | 13:41702235-68468981 bp  13:59638224-59638264 bp | Milk composition  Milk protein percentage | Bouwman et al., 2012  Ning et al., 2017 |
|  | 11 | 0.76737 | 92923032 | 94869558 | 11:93135536-93135576 bp | Milk beta lactoglobulin | Kemper et al., 2018 |
|  | 15 | 0.74747 | 5333494 | 7602018 | 15:5400540-5400580 bp | Milk conjugated linoleic acid content | Strillacci et al., 2014 |
|  | 13 | 0.60848 | 79722466 | 80379008 | 13:79800180-79800220 bp | Milk fatty acids | Buitenhuis et al., 2014 |
|  | 7 | 0.59222 | 38094462 | 43808689 | 7:37028304-38123606 bp  7:43653090-43653130 bp | Milk yield  Milk fat yield | Oliveira et al., 2019  Jiang et al., 2019 |
| Lactation length | 3 | 1.52985 | 46774709 | 47802287 | 3:47777971-47778011 bp  3:46811528-46811568 bp | Milk protein yield  Milk yield | Jiang et al., 2019  Cole et al., 2011 |
|  | 7 | 0.71453 | 29999053 | 31821655 | 7:30261450-30261490 bp | Milk fat yield  Milk protein yield | Meredith et al., 2012 |
|  | 11 | 0.68142 | 23004988 | 25178018 | 11:23501839-23501879 bp | Milk lactose content | Ibeagha-Awemu et al., 2016 |
|  | 14 | 0.65476 | 42745544 | 43884784 | 14:42949997-42950037 bp | Milking speed | Marete et al., 2018 |
|  | 5 | 0.65299 | 83135892 | 84642666 | 5:84318555-84318595 bp  5:83226501-83226541 bp | Milk fat yield  Milk kappa casein percentage | Jiang et al., 2019  Buitenhuis et al., 2014 |
|  | 5 | 0.51863 | 16228162 | 17061285 | 5:16231733-16231773 bp | Milk unglycosylated kappa-casein percentage | Buitenhuis et al., 2014 |
|  | 2 | 1.58296 | 31610105 | 32702939 | 2:31975579-31975619 bp | Milk kappa casein percentage | Buitenhuis et al., 2014 |
|  | 20 | 1.01686 | 14402062 | 17111908 | 20:14003845-14956518 bp  20:13731671-15651653 bp  20:14956498-14956538 bp  20:8021456-24448203 bp | Milk energy yield  Milk fat yield  Milk yield  Milk composition | Lu et al., 2018  Oliveira et al., 2019  Lu et al., 2018  Boumann et al., 2012 |
|  | 13 | 0.85 | 59721374 | 60594871 | 13:59924030-59924070 bp | Milk fatty acid | Knutsen et al., 2018 |
|  | 19 | 0.78498 | 51271259 | 52385253 | 19:51386715-51386755 bp | Milk fatty acids | Benedet et al., 2019 |
|  | 20 | 0.77254 | 50726448 | 52054688 | 20:50969828-50969868 bp | Milk fat percentage | Korkuć et al., 2021 |
|  | 20 | 0.75111 | 32367091 | 34202004 | 20:32878017-32878057 bp  20:32903552-32903592 bp | Milk yield  Milk fat percentage | Jiang et al., 2019 |
|  | 25 | 0.64453 | 4584405 | 5254695 | 25:4649340-4649380 bp | Milk fat yield | Iso-Touru et al., 2016 |
| Dry Period | 20 | 1.09971 | 56367954 | 58279155 | 20:56448993-56449033 bp  20:58219893-58219933 bp | Ketosis  Milk composition | Nayeri et al., 2019  Benedet et al., 2019 |
|  | 4 | 0.67864 | 97693606 | 99193070 | 4:98016955-98016995 bp | Length of productive life  Milk fat yield  Heifer pregnancy rate | Cole et al., 2011 |
|  | 6 | 0.62202 | 42314025 | 43723957 | 6:30995090-45300844 bp | Milk composition | Huang et al., 2012 |
|  | 23 | 0.5461 | 46596800 | 47995741 | 23:47040887-47040927 bp | 305d milk yield | Da cruz et al., 2021 |

**Supplemetary table 6: Relevant QTLs, their positions in base pairs (bp) in cattle chromosome (chr), based on the selected region that explains 0.5% on the windows of 30 adjacent SNPs variance for the reproduction traits**

| Trait | chr | var | Start pos | End pos | QTL POSITION | trait | reference |
| --- | --- | --- | --- | --- | --- | --- | --- |
| Age at first calving | 4 | 0.93971 | 35180959 | 37451963 | 4:37235656-37235696 bp  4:37235656-37235696 bp | First service conception  Conception rate | Galliou et al., 2020  Kiser et al., 2019 |
|  | 8 | 0.90039 | 28792629 | 31306486 | 8:29615869-29615909 bp | Insemination per conception | Kiser et al., 2019 |
|  | 11 | 0.66442 | 18325347 | 19862256 | 11:18221603-19125116 bp | Calving ease | Hoglund et al., 2012 |
|  | 7 | 0.64293 | 20183027 | 21011003 | 7:20209413-20209453 bp | Insemination per conception | Galliou et al., 2020 |
|  | 4 | 0.62651 | 66217790 | 68460261 | 4:63292774-66960121 bp  4:63292774-66960121 bp  4:67145875-67145915 bp | Calving to conception interval  Interval from calving to first estrus  Interval to first estrus after calving | Muller et al., 2017  Liu et al., 2017 |
|  | 14 | 0.58274 | 25869282 | 27092172 | 14:25021594-25986431 bp | Heifer pregnanacy | Junior et al., 2017 |
| Calving Interval | 17 | 0.70878 | 37285288 | 39061918 | 17:38345774-38345814 bp | Interval to first estrus after calving | Hawken et al., 2012 |
|  | 1 | 0.58917 | 138856388 | 140788767 | 1:139950767-139950807 bp  1:140095556-140095596 bp | First service conception  Conception rate  Interval to first estrus after calving | Galliou et al., 2020  Hawken et al, 2012 |
|  | 1 | 0.57603 | 105667081 | 107373821 | 1:105711713-105711753 bp | Conception rate | Kiser et al., 2019 |
|  | 20 | 0.56355 | 18038582 | 20109948 | 20:18394136-18394176 bp | Conception rate | Gaddis et al., 2016 |
| First Service Period | 13 | 1.53812 | 3601539 | 4392350 | 13:3765055-3765095 bp  13:3978334-3978374 bp | Conception rate  Daughter pregnanacy rate | Kiser et al., 2019  Liu et al., 2017 |
|  | 9 | 1.50122 | 46861674 | 48653543 | 9:48483534-48483574 bp | Insemination per conception | Galliou et al., 2020 |
|  | 25 | 1.02212 | 28075219 | 30012018 | 25:28272468-29532547 bp | Interval to first estrus after calving | Liu et al., 2017 |
|  | 4 | 1.01885 | 97548558 | 99039419 | 4:98314913-98314953 bp  4:98016955-98016995 bp | Age at first calving  Daughter pregnancy rate  Length of productive life | Buzanskas etal., 2017  Cole et al., 2011 |
|  | 24 | 0.79599 | 21385194 | 23891473 | 24:22036599-22036639 bp | Interval to first estrus after calving | Hawken et al., 2012 |
|  | 9 | 0.58779 | 48880245 | 50814473 | 9:50208691-50208731 bp | Inseminations per conception | Sahana et al., 2010 |
|  | 16 | 0.57407 | 75216787 | 76956500 | 16:75946910-75946950 bp  16:75305737-75305777 bp | Interval to estrus after calving  Daughter pregnancy rate | Hawken et al., 2012  Cole et al., 2011 |
|  | 1 | 0.57065 | 138856388 | 140788767 | 1:139950767-139950807 bp  1:140095556-140095596 bp | First service conception  Interval to first estrus after calving | Galliou et al., 2020  Hawken et al., 2012 |
|  | 11 | 0.56737 | 1181818 | 3163571 | 11:1901767-1901807 bp | Interval to first estrus after calving | Melo et al., 2019 |
|  | 1 | 0.56299 | 122565644 | 124319995 | 1:122822382-122822422 bp | First service conception | Galliou et al., 2020 |
|  | 6 | 0.56084 | 78178509 | 80635060 | 6:78372791-78372831 bp | First service conception | Galliou et al., 2020 |
