# Supplementary material for: Weighted single step GWAS reveals genomic regions associated with economic traits in Murrah buffaloes
Source: Anim Biotechnol. 2024 Mar 4;35(1):2319622. doi: 10.1080/10495398.2024.2319622 (PMC12674339; doi:10.1080/10495398.2024.2319622)
Supplement: Supplemental Material [file LABT_A_2319622_SM3923.zip › sgwas_production.docx]

**Supplementary table 2: Identification of genes, chromosome and position based on the genetic variance explained by windows of 30 single nucleotide polymorphisms for production traits**

| Traits | chro | var | start | End | genes |
| --- | --- | --- | --- | --- | --- |
| 305day milk yield | 9 | 3.52253 | 12192481 | 15311807 | PCSK1, CHD1, RGMB, RIOK2, LIX1, LNPEP, ERAP1, ERAP2, CAST |
|  | 8 | 2.71385 | 109458876 | 111070457 | CNTNAP2 |
|  | 20 | 1.40315 | 50726448 | 52054688 | NTRK3, AGBL1 |
|  | 20 | 1.37991 | 14110785 | 16626498 | CKMT1A, PPIP5K1, MAP1A, TP53BP1, TUBGCP4, ZSCAN29, ADAL, LCMT2, TGM7, TGM5, MIS18BP1, FANCM, FKBP3, PRPF39, TOGARAM1, KLHL28, FSCB |
|  | 21 | 1.36151 | 39019077 | 39895292 | PTPRG |
|  | 20 | 1.25556 | 6111693 | 6846783 | LOC112581052 |
|  | 16 | 1.00392 | 46251522 | 47885362 | SPON1, RRAS2, COPB1, PSMA1, PDE3B, INSC |
|  | 2 | 0.89372 | 31488482 | 32558439 | PTCHD4, OPN5, ADGRF4, ADGRF2, CD2AP, TNFRSF21, ADGRF1. ADGRF5, MEP1A, ANKRD66, PLA2G7, TDRD6, SLC25A27 |
|  | 2 | 0.88508 | 89761245 | 91263644 | TANC1, DAPL1, PKP4, CCDC148, UPP2, ACVR1 |
|  | 25 | 0.84115 | 4790673 | 5254724 | PNPLA4 |
|  | 13 | 0.79673 | 59545493 | 60180470 | MTUS2, SLC7A1, UBL3 |
|  | 11 | 0.76737 | 92923032 | 94869558 | LHFPL2, SCAMP1, AP3B1, TBCA, OTP, WDR41, PDE8B, ZBED3, AGGF1, CRHBP, S100Z, F2RL1, F2R, IQGAP2, F2RL2 |
|  | 4 | 0.7488 | 53427320 | 55038985 | IGF1, PARPBP, PMCH, NUP37, WASHC3, DRAM1, GNPTAB, SYCP3, CHPT1, MYBPC1, SPIC, ARL1, UTP20, SLC5A8, ANO4 |
|  | 15 | 0.74747 | 5333494 | 7602018 | PSK2, ATP6V0D2, WWP1, RMDN1, CPNE3, MMP16 |
|  | 13 | 0.60848 | 79722466 | 80379008 | OLFM4 |
|  | 7 | 0.59222 | 38094462 | 43808689 | ADGRL3 |
|  | 8 | 0.59041 | 50559358 | 53165190 | CFTR, ASZ1, WNT2, ST7, CAPZA2, MET, CAV1, CAV2, TES, TFEC |
| Lactation length | 3 | 1.52985 | 46774709 | 47802287 | CCL1 |
|  | 4 | 1.08357 | 123353383 | 124243430 | TRIM67, GNPAT, EXOC8, SPRTN, EGLN1, TSNAX, DSC1 |
|  | 19 | 0.8661 | 7593833 | 8773828 | ARHGEF28, UTP15, ANKRA2, BTF3, FOXD1, TMEM174, TMEM171, FCHO2, TNPO1 |
|  | 7 | 0.71453 | 29999053 | 31821655 | ADAMTS3, NPFFR2, GC, SLC4A4, DCK, MOB1B, GRSF1, RUFY3, UTP3, JCHAIN,ENAM, AMBN, AMTN, MUC7 |
|  | 3 | 0.69837 | 171177232 | 172345492 | BRINP1 |
|  | 11 | 0.68142 | 23004988 | 25178018 | RAD51B, ZFYVE26, RDH12, RDH11, VTI1B, ARG2, PIGH, PLEKHH1, TMEMM229B, PLEK2,EIF2S1, ATP6V1D, MPP5,FAM71D, GPHN, CCDC196 |
|  | 4 | 0.65761 | 52300866 | 53171692 | SCLT8D2, TDG, HSP90B1, NT5DC3, STAB2 |
|  | 14 | 0.65476 | 42745544 | 43884784 | PAX1, NKX2-2, NKX2-4, XRN2, KIZ, RALGAPA2 |
|  | 5 | 0.65299 | 83135892 | 84642666 | CTSC, RAB38, TMEM135, FZD4,PRSS23, ME3 |
|  | 5 | 0.51863 | 16228162 | 17061285 | RGL1, APOBEC4, ARPC5, NCF2, SMG7, NMNAT2, LAMC1, LAMC2 |
| Total milk yield | 9 | 2.1929 | 10946925 | 12495803 | LOC102398863 |
|  | 8 | 1.76233 | 109458876 | 111070457 | CNTNAP2 |
|  | 2 | 1.58296 | 31610105 | 32702939 | OPN5, ADGRF4, ADGRF2, CD2AP, TNFRSF21, ADGRF1, ADGRF5, MEP1A, ANKRD66, PLA2G7, TDRD6, RCAN2 |
|  | 20 | 1.03723 | 6111693 | 6846783 | LOC112580870 |
|  | 20 | 1.01686 | 14402062 | 17111908 | TGM5, MIS18BP1, FANCM, FKBP3, PRPF39, KLHL28, FSCB |
|  | 13 | 0.85 | 59721374 | 60594871 | ATP12A, RNF17, CENPJ, PAPP4, MPHOSPH8, PSPC1, ZMYM5, GJA2, GJB2, CRYL1, IFT88, IL17D, EEF1AKMT1, XPO4, LATS2, SAP18, SKA3, MRPL57,MKU2 |
|  | 19 | 0.78498 | 51271259 | 52385253 | CDH12 |
|  | 20 | 0.77254 | 50726448 | 52054688 | AGBL1, NTRK3 |
|  | 20 | 0.75111 | 32367091 | 34202004 | NOVA1 |
|  | 25 | 0.64453 | 4584405 | 5254695 | STS, PNPLA4 |
|  | 4 | 0.62073 | 53869201 | 55319682 | PARPBP, PMCH, NUP37, WASHC3, DRAM1, GNPTAB, SYCP3, CHPT1, MYBPC1, SPIC, ARL1, UTP20, ANO4 |
| Dry period | 18 | 2.70048 | 12334366 | 13937362 | FBX031, MAP1LC3B, ZCCHC14, JPH3, KLHDC4, SLC7A5, CA5A, BANP, ZNF469, ZFPM1, ZC3HH18, IL17C, MVD, SNAI3, RNF166, CTU2, PIEZO1, CDT1, APRT, CALNS, TRAPPC2L, PABPN1L, CBFA2T3, ACSF3, CDH15, SLC22A31, ANKRD11 |
|  | 5 | 1.44895 | 2549051 | 3382482 | PTPRC |
|  | 20 | 1.09971 | 56367954 | 58279155 | MCTP2 |
|  | 6 | 1.04847 | 26532208 | 27219535 | IGSF3, CD58, ATP1A1, MAB21L3, SLC22A15 |
|  | 7 | 0.71199 | 100403992 | 101534282 | MTTP, TRMTI0A, ADH4, METAP1, EIF4E, TSPAN5 |
|  | 4 | 0.67864 | 97693606 | 99193070 | BTG1, DCN |
|  | 6 | 0.62202 | 42314025 | 43723957 | GPR88, CDC14A, RTCA, DBT, LRRC39, TRMT13, SASS6, MFSD14A, AGL |
|  | 23 | 0.5461 | 46596800 | 47995741 | FOXI2, CLRN3, PTPRE, MKI67 |
