# Supplementary material for: Weighted single step GWAS reveals genomic regions associated with economic traits in Murrah buffaloes
Source: Anim Biotechnol. 2024 Mar 4;35(1):2319622. doi: 10.1080/10495398.2024.2319622 (PMC12674339; doi:10.1080/10495398.2024.2319622)
Supplement: Supplemental Material [file LABT_A_2319622_SM3923.zip › Wssgwas_reproduction.docx]

**Supplementary table 3: Identification of genes, chromosome and position based on the genetic variance explained by windows of 30 single nucleotide polymorphisms for reproduction traits**

| Trait | chr | var | Start pos | End pos | gene |
| --- | --- | --- | --- | --- | --- |
| Age at first calving | 14 | 2.2026 | 44928887 | 46500564 | SLC2483, SCP2D1, DTD1, SMIM26, SEC23B, RBBP9, RRBP1, DSTN, BFSP1, PC5K2, POLR3F, DZANK1, KAT14, PET117, OVOL2, MGME1, SNX5, BANF2 |
|  | 3 | 1.92581 | 166249601 | 167454677 | AKNA, WHRN,ATP6V1G1,TMEM268,TEX48, TNFSF8,TNFSF15, TNC |
|  | 14 | 1.74054 | 41905286 | 42536655 | CD93, THBD, FOXA2 |
|  | 19 | 1.04672 | 56372926 | 57379623 | RETREG1, ZNF622, MARCH11, FBXL7 |
|  | 6 | 1.01811 | 1468005 | 2888860 | DUSP27, GPA33, MAEL, ILDR2, TADA1, POGK, FAM78B, UCK2 |
|  | 4 | 0.93971 | 35180959 | 37451963 | BCAT1, LRMP, CASC1, ETFRF1, KRAS, LMNTDI, RASSF8, BHLHE41, SSPN, ITPR2, INTS13, FGFR1QP2, TM7SF3 |
|  | 8 | 0.90039 | 28792629 | 31306486 | ITGB8, ABCB5, SP8, SP4, DNAH11, CDCA7L, RAPGEF5, IL6, TOMM7 |
|  | 11 | 0.66442 | 18325347 | 19862256 | HEATR4, RIOX1, NUMB, PAPLN, PSEN1, RBM25, ZFYVEI, DCAF4, DPF3, RGS6 |
|  | 7 | 0.64293 | 20183027 | 21011003 | TMEM150C, ENOPH1, HNRNPDL, HNRNPD |
|  | 1 | 0.63854 | 197338299 | 198731327 | ANKRD28, GALNT15, DPH3, OXNAD1, RFTN1, DAZL, PLCL2, TBC1D5 |
|  | 3 | 0.63453 | 152863651 | 154511480 | PLPPR1, MRPL50, ZNF189, ALDOB, TMEM246, RNF20, GRIN3A, PPP3R2 |
|  | 4 | 0.62651 | 66217790 | 68460261 | SLC16A7, FAM19A2 |
|  | 1 | 0.5908 | 153632913 | 154835593 | RSRC1, SHOX2, VEPH1, PTX3 |
|  | 14 | 0.58274 | 25869282 | 27092172 | STX16, NPEPL1, NELFCD, CTSZ, TUBB1, ATP5F1E, PRELID3B, ZNF831, EDN3, PHACTR3, SYCP2, PPP1R3D, FAM217B, CDH26 |
|  | 9 | 0.55155 | 103631489 | 104267102 | KLF2,EPSI5L1, CALR3, CHERP, SLC35E1, MED26, SMIM7, TMEM38A, NWD1, SIN3B, F2RL3, CPAMD8 |
|  | 5 | 0.547 | 101157914 | 102046184 | E2F8, CSRP3, ZDHH, C13, PTPN5, IGSF22, TMEM86A, TRNA-UUC, SPTY2D1, SPTY2D1OC, UEVLD, TSG101, GTF2H1, HPS5 |
| Calving interval | 25 | 1.34335 | 138740090 | 142909023 | NSDL, ZNF185, PNMA5, CETN2, GABR, VAMP7, SPRY3, TMLHE, CLIC2, RAB39B, VBP1, BRCC3, CMC4, MTCP1, FUNDC2, F8, F8A1, SMIM9, TRNAY-AUA, MPP1, DKC1, GAB3, ZNF275, PNMA6E, ZFP92, TREX2, HAUS7, BGN |
|  | 17 | 0.70878 | 37285288 | 39061918 | TRPC3, KIAA1109, IL2, IL21, BBS12, FGF2, NUDT6, SPATA5, SPRY1 |
|  | 1 | 0.60196 | 43319702 | 45411355 | MYOM2, KBTBD11, ARHGEF10, CLN8, DLGAP2, ERICH1, CLIC6 |
|  | 1 | 0.58917 | 138856388 | 140788767 | SPATA16, ECT2, NCEH1, TNFSF10, GHSR, FNDC3B, TMEM212, PLD1, TNIK |
|  | 25 | 0.58165 | 17881183 | 20207971 | SH3KBP1, BCLAF3, MAP7D2, EIF1AX, RPS6KA3, CNKSR2, KLHL34, SMPX, MBTPS2, SMS, PHEX |
|  | 1 | 0.57603 | 105667081 | 107373821 | LSAMP |
|  | 20 | 0.56355 | 18038582 | 20109948 | LRFN5 |
|  | 9 | 0.5603 | 48566301 | 49752773 | SLC36A2, GM2A, CCDC69, ANXA6, TNIP1, GPX3, ZNF300, SMIM3, DCTN4, RBM22, MYOZ3, SYNPO, NDST1, RPS14, CD74, TCOF1, ARSI, CAMK2A, SLC6A7, CDX1, PDGFRB |
|  | 3 | 0.51932 | 77544036 | 79356632 | LINGO2 |
| First service period | 13 | 1.53812 | 3601539 | 4392350 | FAM155A |
|  | 9 | 1.50122 | 46861674 | 48653543 | NMUR2, GLRA1, G3BP1, ATOX1, SPARC, FAT2, SLC36A2 |
|  | 25 | 1.02212 | 28075219 | 30012018 | IL1RAPL1, NR0B1,GK, TAB3 |
|  | 4 | 1.01885 | 97548558 | 99039419 | EEA1, PLEKHG7, BTG1 |
|  | 15 | 0.87709 | 65602299 | 67473313 | MTSS1, ZHX1, ATAD2, WDYHV1, FBX032, KLHL38, ANXA13, FAM91A1,FER1L6, TMEM65, TRMT12, RNF139, TATDN1, NDUFB9, ZNF572, SQLE, WASHC5, NSMCE2 |
|  | 3 | 0.8241 | 77544036 | 79356632 | LINGO2 |
|  | 24 | 0.79599 | 21385194 | 23891473 | COG7, SCNN1B, SCNN1G, USP31, HS3ST2, OTOA, METTL9, IGSF6, CDR2, POLR3E, EEF2K, SDR42E2, VWA3A, MOSMO, PDZD9, CRYM, ANKS4B, ZP2, TMEM159, DNAH3, LYRM1, DCUN1D3 |
|  | 4 | 0.69307 | 128504931 | 131608583 | EDARADP, LGALS8, HEATR1, ACTN2, MTR, RYR2, ZP4, CHRM3 |
|  | 16 | 0.63842 | 81456361 | 83903218 | CASP1, GRIA4, KBTBD3, AASDHPPT, ANKRD49, MRE11, GPR83, IZUMO1R |
|  | 3 | 0.60358 | 9539349 | 10845904 | SOCS3, PGS1, DNAH17,CYTH1, USP36, TIMP2, CEP295NL, CANT1, C1QTNF1, ENGASE, RBFOX3, ENPP7, CBX2, CBX8, CBX4, TBC1D16 |
|  | 9 | 0.58779 | 48880245 | 50814473 | TNIP2, GPX3, ZNF300, SMIM3, DCTN4,RBM22, MYOZ3, SYNPO, NDST1, RPS14, CD74, TCOF1, ARS1, CAMK2A, SLC6A7, CDX1, PDGFRB, CSF1R, HMGXB3, SLC26A2, PDE6A, PPARGC1B, ARHGEF37, CSNK1A1, IL17B, PCY0X1L, GRPEL2, AFAP1L1, ABLIM3, SH3TC2 |
|  | 16 | 0.57407 | 75216787 | 76956500 | CNTN5, ARHGAP42 |
|  | 1 | 0.57065 | 138856388 | 140788767 | SPATA16, ECT2, NCEH1, TNFSF10, GHSR, FNDC3B, TMEM212, PLD1, TNIK |
|  | 14 | 0.56889 | 55711355 | 57627146 | SEPHS1,PHYH, MCM10, UCMA, OPTN, FZD8, GJD4, GAD2, MYO3, GPR158 |
|  | 11 | 0.56737 | 1181818 | 3163571 | CALM1, NRDE2, PSMC1, KCNK13, TDP1, EFCAB11, FOXN3, TTC8, EML5, ZC3H14, PTPN21, SPATA7 |
|  | 1 | 0.56299 | 122565644 | 124319995 | TPRG1, LPP |
|  | 6 | 0.56084 | 78178509 | 80635060 | PDE4B, LEPROT, DNAJC6, AK4, JAK1, RAVER2, CACHD1, UBE2U |
|  | 14 | 0.54822 | 10396091 | 11510974 | RIMS4, KCNK15, WISP2, ADA, PKIG, SERINC3, TTPAL, HNF4A, R3HDML, FITM2, GDAP1L1, OSER1, JPH2, TOX2, GTSF1L, MYBL2, IFT52, SGK2, L3MBTL1 |
